# Visibility evaluation of gastric epithelial neoplasm of fundic gland mucosa lineage using texture and color enhancement imaging

**DOI:** 10.1002/deo2.70110

**Published:** 2025-04-08

**Authors:** Hisanori Utsunomiya, Hiroya Ueyama, Tsutomu Takeda, Shunsuke Nakamura, Yasuko Uemura, Tomoyo Iwano, Momoko Yamamoto, Ryota Uchida, Daiki Abe, Shotaro Oki, Nobuyuki Suzuki, Atsushi Ikeda, Yoichi Akazawa, Kumiko Ueda, Mariko Hojo, Shuko Nojiri, Takashi Yao, Akihito Nagahara

**Affiliations:** ^1^ Department of Gastroenterology Juntendo University School of Medicine Tokyo Japan; ^2^ Medical Technology Innovation Center Juntendo University School of Medicine Tokyo Japan; ^3^ Department of Human Pathology Juntendo University Graduate School of Medicine Tokyo Japan; ^4^ Department of Pathophysiological Research and Therapeutics for Gastrointestinal Disease Juntendo University School of Medicine Tokyo Japan

**Keywords:** gastric adenocarcinoma of fundic‐gland mucosa type, gastric adenocarcinoma of fundic‐gland type, gastric epithelial neoplasm of fundic‐gland mucosa lineage, oxyntic gland adenoma, texture and color enhancement imaging

## Abstract

**Objectives:**

Recently, the incidence of Helicobacter pylori‐uninfected gastric cancers, such as gastric epithelial neoplasm of fundic‐gland mucosa lineage (GEN‐FGML), has increased with the widespread use of eradication therapy. Because the detection and endoscopic diagnosis of GEN‐FGML are difficult, an effective observation method in screening endoscopy is required. We investigated whether texture and color enhancement imaging (TXI) improved the visibility of GEN‐FGML compared with white light imaging (WLI).

**Methods:**

In this single‐center prospective clinical study, 50 GEN‐FGML lesions (35 patients) treated at our hospital between October 2020 and June 2023 were analyzed. The endoscopic images of GEN‐FGML obtained using WLI, TXI mode 1 (TXI‐1), TXI mode 2 (TXI‐2), and narrow‐band imaging were compared by 10 endoscopists. We analyzed the visibility score and inter‐rater reliability (intraclass correlation coefficient and conducted an objective evaluation based on L* a* b* color values and the color difference (ΔE*) in the CIE LAB color space system.

**Results:**

Histologically, GEN‐FGML was classified as gastric adenocarcinoma of fundic‐gland type (*n* = 45) and gastric adenocarcinoma of fundic‐gland mucosa type (*n* = 5). The total visibility score for all endoscopists was significantly higher for TXI than for WLI (*p* < 0.01); and for TXI‐1 than for TXI‐2 (*p* < 0.01). The intraclass correlation coefficients for TXI‐1 and TXI‐2 were “almost perfect” and “substantial,” respectively, for all endoscopists. ΔE* was significantly higher for TXI than for WLI (*p* < 0.01).

**Conclusions:**

TXI improved the visibility of GEN‐FGML for all endoscopists compared with WLI when evaluated subjectively and objectively.

## INTRODUCTION

The development of gastric cancer has been linked to histological changes caused by *Helicobacter pylori* infection, such as atrophic gastritis and intestinal metaplasia.^1^, ^2^ In Japan, most patients with gastric cancer are infected with *H. pylori*, with many receiving *H. pylori* eradication therapy, whereas *H. pylori*‐uninfected gastric cancer (HPUGC) is relatively uncommon (0.42%–2.5% of gastric cancers).^3^, ^4^, ^5^, ^6^, ^7^ Recently, the incidence of *H. pylori*‐positive patients has declined with the increased use of *H. pylori* eradication therapy, with the incidence of HPUGC consequently rising.^5^

In recent years, many HPUGC lesions, such as oxyntic gland adenoma (OGA), gastric adenocarcinoma of fundic‐gland type (GA‐FG), and gastric adenocarcinoma of fundic‐gland mucosa type (GA‐FGM), tend to differentiate into a fundic gland cell, foveolar epithelial cell, or mucous neck cell lineage. These cancers are collectively termed gastric epithelial neoplasm of fundic‐gland mucosa lineage (GEN‐FGML).^8^, ^9^ OGA and GA‐FG are listed as rare gastric neoplasias in the 5th edition of the World Health Organization (WHO) Classification of Tumours, 2019.^10^ We previously described the endoscopic features of GEN‐FGML.^11^, ^12^, ^13^, ^14^ In WLI, the most common features of OGA/GA‐FG were submucosal tumor shape, whitish color, dilated vessels with branching architecture, and background mucosa without atrophic change.^12^ However, GEN‐FGML is difficult to detect because its endoscopic and clinicopathological characteristics differ from those of typical gastric cancer.^11^, ^12^, ^13^, ^14^ Therefore, an effective method for detecting GEN‐FGML in screening endoscopy is required.

In this context, a new image enhancement technology called texture and color enhancement imaging (TXI; Olympus Medical Systems Corporation), which optimizes structure (texture) enhancement, color tone, and brightness correction, was developed in 2020. TXI easily highlights differences in mucosal color and structure,^15^ permitting the easy recognition of structural changes and slight color changes in the image and thereby facilitating tumor detection. Several studies described the utility of TXI for detecting and diagnosing early gastric cancer (EGC), mucosal atrophy, and gastric neoplasms.^16^, ^17^, ^18^, ^19^, ^20^, ^21^, ^22^, ^23^, ^24^ However, the usefulness of TXI for detecting GEN‐FGML has not been investigated. Therefore, we investigated whether TXI improved the visibility of GEN‐FGML versus white light imaging (WLI).

## METHODS

### Study design and patients

In this single‐center prospective clinical study, patients who underwent esophagogastroduodenoscopy (EGD) with WLI, TXI, or narrow‐band imaging (NBI) between October 2020 and June 2023 at our hospital were enrolled consecutively.

We analyzed visibility as a subjective variable and differences in pigmentation as an objective variable. Patients were excluded if it was difficult to evaluate their endoscopic images because of mucus or erosions and immunohistochemical staining was not fully evaluated. The definition of *H. pylori* infection is provided in Figure S1.

### Endoscopic procedure

Endoscopy was performed using the GIF‐XZ1200 endoscopes and EVIS X1 CV‐1500 Video System Center (both from Olympus Medical Systems Corporation). All endoscopic examinations were performed by three expert endoscopists (YA, TT, HU), who assessed and discussed WLI images until consensus was achieved. Most patients were outpatients who underwent endoscopy with mild sedation using midazolam. Images were obtained from the server in the JPEG format with no loss of quality. Each image was approximately 100 kB in size, the pixel array was 640 × 510, and 24‐bit color was applied.

### Histopathological classification of GEN‐FGML

According to the histopathological classification, GEN‐FGML was classified as OGA, GA‐FG, or GA‐FGM.^8^ The diagnostic criteria are provided in Figure S2. In this study, so‐called OGA, at least for a lesion presenting histological features similar to GA‐FG, should be regarded as an intramucosal phase of GA‐FG. In addition, according to the mucosal architecture of the foveolar epithelium and fundic gland, GA‐FGM was classified into three subtypes (Figure S3).^8^

All histology and immunohistochemical staining results were evaluated by one pathologist (Takashi Yao) specializing in the pathophysiology of the gastrointestinal tract. This pathologist is also one of the authors of the OGA section in the WHO Classification of Tumors, 2019.

### Visibility evaluation of GEN‐FGML

Ten endoscopists, including five experts (Yoichi Akazawa, Atsushi Ikeda, Daiki Abe, Shotaro Oki, and Nobuyuki Suzuki) and five trainees (Ryota Uchida, Hisanori Utsunomiya, Tomoyo Iwano, Momoko Yamamoto, and Yasuko Uemura) used WLI, TXI mode 1 (TXI‐1), TXI mode 2 (TXI‐2), and NBI to evaluate endoscopic images of GEN‐FGML. The experts consisted of gastroenterologists board‐certified by the Japan Gastroenterological Endoscopy Society with at least 5 years of experience in endoscopy and prior experience using the TXI system. Trainees were uncertified, and they had fewer than 5 years of experience in endoscopy. Using representative TXI and NBI images, the visibility of gastric lesions was evaluated in reference to WLI‐based endoscopy. WLI–TXI‐1, WLI–TXI‐2, and WLI–NBI images were displayed side‐by‐side. Anonymous images lacking any identifying clinical data or the date of imaging were presented to the endoscopists, who evaluated visibility using the following scale: 1, decreased; 2, somewhat decreased; 3, equivalent; 4, somewhat improved, and 5, improved. The scores of the endoscopists were summed to calculate the total score, which was categorized as follows: ≤20, decreased visibility; 21–39, equivalent visibility; and ≥40, improved visibility. In addition, the intraclass correlation coefficient (ICC) together with its 95% confidence interval (CI) was determined as a measure of interrater reliability. ICCs are suitable when two or more coders are used in studies. The ICC was categorized as follows: 1.0, perfect; 0.81–0.99, almost perfect; 0.61–0.80, substantial; 0.41–0.60, moderate; 0.21–0.40, fair; and ≤0.20, slight.^25^, ^26^

### Objective evaluation in color difference

In the objective evaluation, the images were analyzed and scored according to the L* a* b* color values (L* = light/dark; a* = red/green; b* = yellow/blue) in the CIE LAB color space system^27^ using Adobe Photoshop CC 2019 as previously described.^28^ A region of interest (ROI, 40 × 40 pixels) was set to encompass the lesion and background mucosa. The ROI covered the same area in all four images for each lesion. The average color values (*L, a, b*) in an ROI were calculated from the histogram panel. *L, a*, and *b* were the color values in the Lab color unit of Adobe Photoshop, and they were transformed to L*, a*, and b* color values in CIE LAB as follows: L* = *L*/256 × 100; a* = *a* − 128; and b* = *b* − 128.^29^, ^30^ The color difference (ΔE* = [(ΔL*)^2^ + (Δa*)^2^ + (Δb*)^2^] 1/2) of the pixel values based on the L* a* b* color spaces within the ROI was analyzed to evaluate the visibility of each color image.

### Statistical analysis

Differences between trainees and experts for visibility scores and differences among TXI‐1, TXI‐2, and NBI for visibility scores were examined using the Mann–Whitney U test. *p *< 0.017 as determined by Bonferroni's multiple comparison test was considered significant. Differences between each image for the L*, a*, b* color values, and ΔE* were examined using the Mann–Whitney U test. *p* < 0.0083 as determined by Bonferroni's multiple comparison test was considered significant.

To test for inter‐rater reliability, we used ICCs. The ICC is commonly used to assess inter‐rater reliability for ordinal, interval, and ratio variables. Intra‐class correlation coefficients are suitable for when two or more coders are used in studies.^31^ SPSS for Windows, version 28.0 (IBM, Armonk, NY, USA) was used for statistical analyses.

## RESULTS

### Patient characteristics

This study enrolled 35 patients with 50 lesions. The GEN‐FGMLs were categorized as OGA/GA‐FGs (*n* = 45) and GA‐FGMs (*n* = 5). The clinicopathological findings of each type are presented in Table 1. Most OGA/GA‐FGs were whitish lesions, and they were macroscopically classified as the slightly elevated type (0‐IIa) or flat type (0‐IIb). GA‐FGMs exhibited varied coloration, and they were classified as type 1 (*n* = 3) or type 3 (*n* = 2). Table 2 presents the immunohistochemical findings of GEN‐FGML, and the results were consistent with its previously reported characteristics.^8^, ^9^

**TABLE 1:** Clinicopathological findings of gastric epithelial neoplasm of fundic‐gland mucosa lineage.

	OGA/GA‐FG	GA‐FGM
**Patients (*n* = 35)**	*n* = 30	*n* = 5
Sex (male/female)	17/13	3/2
Age (mean), years (range)	72.0 (36–89)	58.0 (32–76)
Survival periods (mean), months (range)	12 (1–47)	15 (2–16)
**Lesions (*n* = 50)**	*n* = 45	*n* = 5
Endoscopic findings		
Tumor size (mean), mm (range)	4.0 (1‐12)	6.0 (4‐8)
Location (U/M/L)	35/10/0	3/2/0
Coloration (reddish/whitish)	9/36	2/3
Macroscopic type (0‐I/0‐IIa/0‐IIb/0‐IIc)	3/24/14/4	1/2/1/1
*H. pylori* infection status (uninfected/post‐eradication/positive)	37/8/0	5/0/0
Subtypes of GA‐FGM (type 1/type 2/type 3)	‐	3/0/2
Pathological findings		
Depth of invasion (M/SM1/ SM2)	26/18/1	2/2/1
Distance of SM invasion (mean), µm (range)	175 (50–600)	450 (300–1000)
Lymphatic invasion (+)	0/45	0/5
Venous invasion (+)	0/45	0/5
Horizontal margin (+)	1/45	0/5
Vertical margin (+)	1/45	0/5

Abbreviations: 0‐I, elevated type; 0‐IIa, slightly elevated type; 0‐IIb, flat type; 0‐IIc, slightly depressed type; GA‐FG, gastric adenocarcinoma of fundic‐gland type; GA‐FGM, gastric adenocarcinoma of fundic‐gland mucosa type; GEN‐FGML, gastric epithelial neoplasm of fundic‐gland mucosa lineage; L, lower body; M, middle body; M, mucosal layer; OGA, oxyntic gland adenoma; SM, submucosal layer; U, upper body.

**TABLE 2:** Immunohistochemical findings of gastric epithelial neoplasm of fundic‐gland mucosa lineage.

	OGA/GA‐FG (*n* = 45)	GA‐FGM (*n* = 5)
**Cell differentiation**		
MUC5AC (+)	0％ (0/45)	100％ (5/5)
MUC6 (+)	87％ (39/45)	100％ (5/5)
pepsinogen‐1 (+)	100％ (45/45)	100％ (5/5)
H+/K+ ATPase (+)	73％ (33/45)	60％ (3/5)
MUC2 (+)	0％ (0/45)	0％ (0/5)
CD10 (+)	0％ (0/45)	0％ (0/5)
**Mucin phenotype**		
Gastric phenotype	100％ (45/45)	100％ (5/5)
Gastrointestinal phenotype	0％ (0/45)	0％ (0/5)
Intestinal phenotype	0％ (0/45)	0％ (0/5)
Unclassified phenotype	0％ (0/45)	0％ (0/5)
p53 overexpression	0％ (0/45)	0％ (0/5)
Ki‐67 labeling index, mean (range)	2.0 (1‐10)	5.0 (1‐20)

Abbreviations: GA‐FG, gastric adenocarcinoma of fundic‐gland type; GA‐FGM, gastric adenocarcinoma of fundic‐gland mucosa type; GEN‐FGML, gastric epithelial neoplasm of fundic‐gland mucosa lineage; OGA, oxyntic gland adenoma.

### Case presentation

#### Case 1: Gastric adenocarcinoma of GA‐FG

**FIGURE 1:**
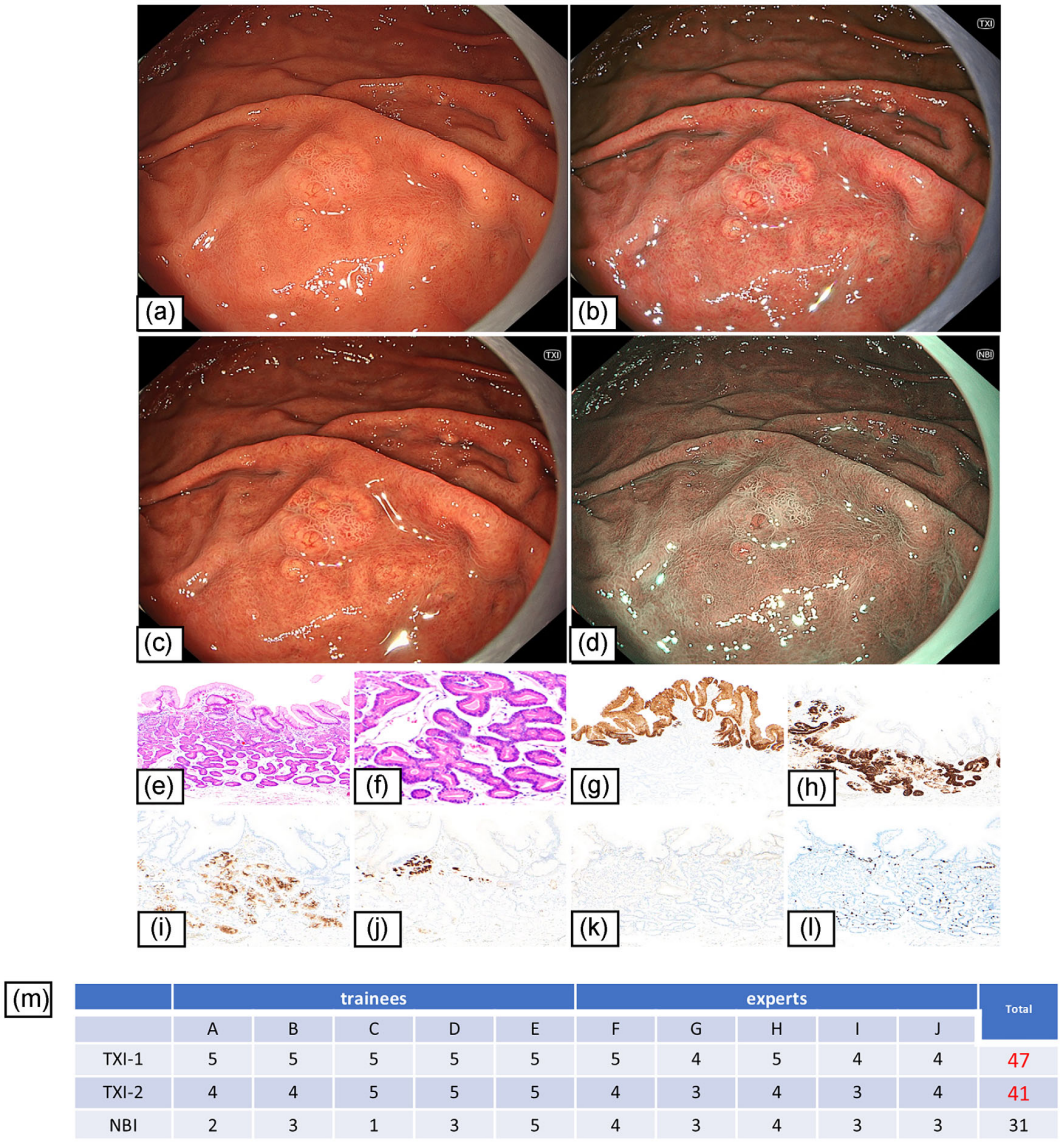
Case 1: Endoscopic images of GA‐FG using white light imaging (WLI), texture and color enhancement imaging (TXI), and narrow‐band imaging (NBI). This is a representative case in which both TXI‐1 and TXI‐2 showed improved visibility of demarcation. (a) Wh showed a whitish and elevated lesion, 18 mm in size, with SMT‐like elevation at the greater curvature in the upper third of the stomach. Background gastric mucosa indicated *H.pylori* uninfected status. The demarcation line was indistinct. (b) TXI‐1 improved the visibility of the demarcation line and the dilated vessels with branched architecture which was one of the endoscopic characteristics of GA‐FG. (c) TXI‐2 also improved the visibility of the demarcation line and the dilated vessels with branched architecture. (d) NBI did not improve the visibility of the demarcation line and dilated vessels with branched architecture. (e, f) HE stains; gastric adenocarcinoma resembling fundic‐gland cells with pale eosinophilic cytoplasm was located from beneath the surface to the deep area (SM 400 µm). (g–l) Immunohistochemical findings. (g) MUC5AC (a marker for gastric foveolar epithelial cells). Negative. (h) MUC6 (a marker for gastric mucous neck cells). Positive. (i) Pepsinogen‐1 (a marker for chief cells). Positive. (j) H+K+ATPase (a marker for parietal cells). Partially positive. (k) p53 overexpression. Negative. (l) Ki‐67 Labeling index 5%. Pathological diagnosis; U, Gre, Type 0‐IIa, 9 × 9 mm, Adenocarcinoma of fundic‐grand type, pT1b (SM1), INFa, pUL0, Ly0, V0, pHM0, and pVM0. (m) The visibility score showed improved visibility (≥40) for TXI‐1 and TXI‐2.

The visibility score for all endoscopists revealed improved visibility (≥40) for TXI. Specifically, TXI improved the visibility of the demarcation line and the dilated vessels with branched architecture, which is one of the endoscopic characteristics of GA‐FG (Figure 1b,c). NBI did not improve the visibility of the demarcation line or the vessels (Figure 1d).

#### Case 2: Gastric adenocarcinoma of GA‐FGM

**FIGURE 2:**
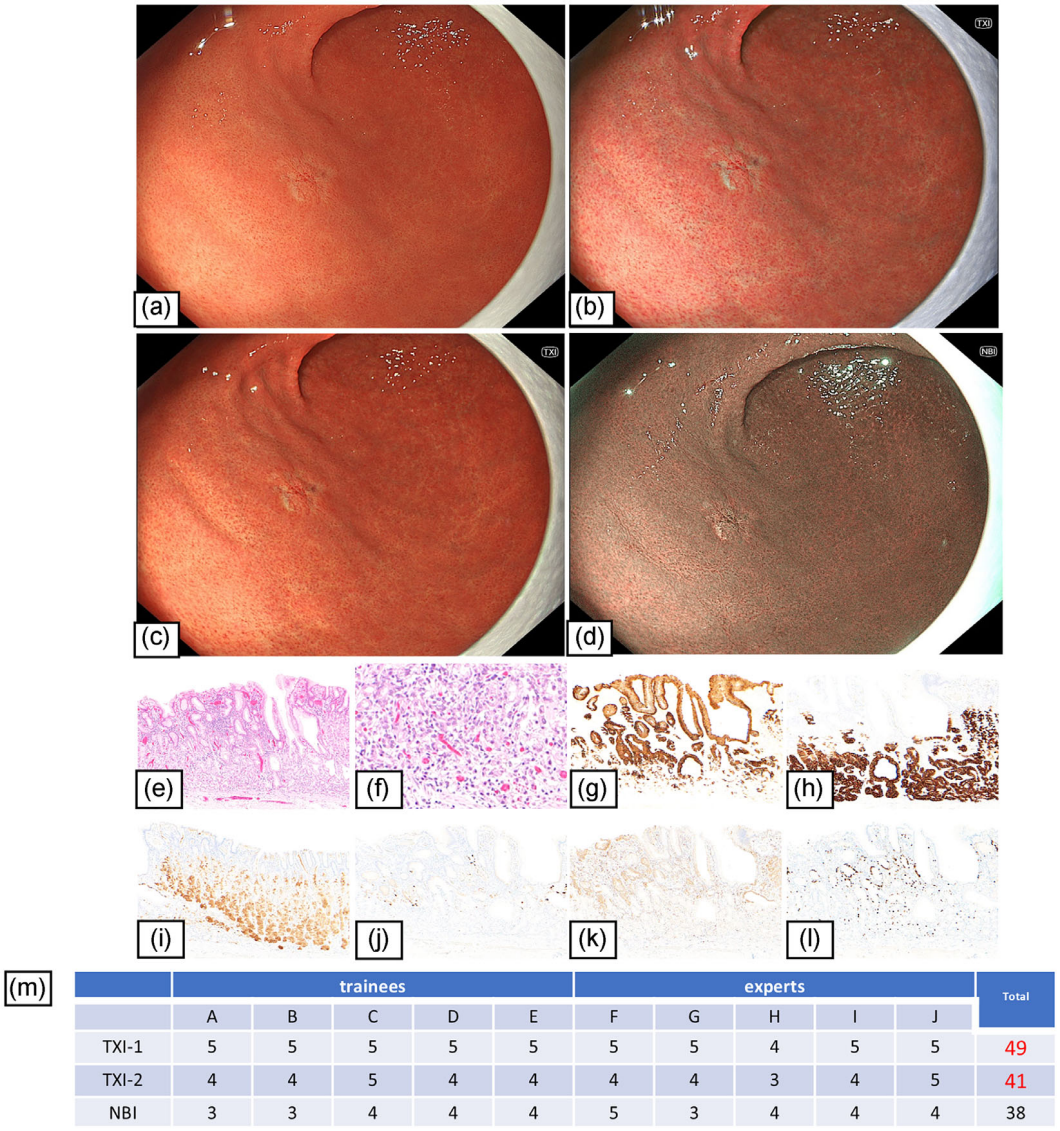
Case 2: Endoscopic images of Type 3 GA‐FGM using white light imaging, texture and color enhancement imaging (TXI), and narrow‐band imaging (NBI). This is a representative case in which both TXI‐1 and TXI‐2 showed improved visibility of demarcation. (a) White light imaging showed a whitish depressed lesion, 10 mm in size, at the greater in the middle third of the stomach. (b) TXI‐1 improved the visibility of the demarcation line and emphasized the whitish area in this lesion. (c) TXI‐2 also improved the visibility of the demarcation line and emphasized the whitish area in this lesion. (d) NBI also improved the visibility of the demarcation line. (e, f) HE stain; the surface of the lesion was covered with normal foveolar epithelium, whereas the deep area of the tumor resembling mucous neck cells and chief cells showed irregular branching and dilatation. (g–l) Immunohistochemical findings. (g) MUC5AC (a marker for gastric foveolar epithelial cells). Positive. (h) MUC6 (a marker for gastric mucous neck cells). Positive. (i) Pepsinogen‐1 (a marker for chief cells). Positive. (j) H+K+ATPase (a marker for parietal cells). Partially positive. (k) p53 overexpression. Negative. (l) Ki‐67 Labeling index 5%. Pathological diagnosis; M, Gre, Type 0‐IIc, 8 × 5 mm, Adenocarcinoma of fundic‐grand mucosa type, pT1a (M), pUL0, Ly0, V0, pHM0, pVM0, and ple. (m) The visibility score showed improved visibility (≥40) for TXI‐1 and TXI‐2.

The visibility score for all endoscopists indicated improved visibility (≥40) for TXI. TXI improved the visibility of the demarcation line and emphasized the whitish area in this lesion (Figure 2b,c). NBI also improved the visibility of the demarcation line (Figure 1d).

### Visibility scores

**FIGURE 3:**
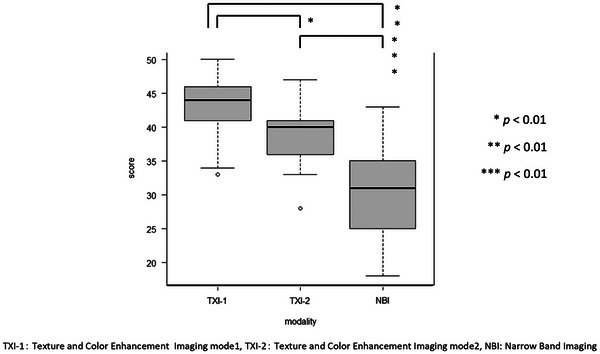
The results of comparison between texture and color enhancement imaging (TXI)‐1, TXI‐2, and narrow‐band imaging (NBI) on visibility scores. Visibility scores were significantly higher in the order of TXI‐1 > TXI‐2 > NBI.

The visibility scores of experts, trainees, and all endoscopists using TXI and NBI compared with WLI are presented in Table 3. For all endoscopists, the total visibility scores for TXI‐1, TXI‐2, and NBI were 44.0, 40.0, and 31.0, respectively. The scores for experts were 21.0, 18.5, and 15.0, respectively, whereas those for trainees were 23.0, 21.0, and 14.5, respectively. Using TXI, the visibility score was significantly higher for trainees than for experts (*p* < 0.01). The total visibility score for all endoscopists was significantly higher for TXI‐1 than for TXI‐2 (*p* < 0.01) and significantly higher for TXI than for NBI (*p *< 0.01, Figure 3).

**TABLE 3:** Visibility scores of experts, trainees, and all endoscopists.

	Score			*p*‐value
Modality	All endoscopists (*n* = 10)	Experts (*n* = 5)	Trainees (*n* = 5)	Experts vs. Trainees
TXI‐1	44.0 ± 3.9	21.0 ± 2.2	23.0 ± 2.0	<0.01
TXI‐2	40.0 ± 3.6	18.5 ± 2.1	21.0 ± 1.9	<0.01
NBI	31.0 ± 5.9	15.0 ± 2.8	14.5 ± 3.4	0.145

Abbreviations: NBI, narrow‐band imaging; TXI‐1, texture and color enhancement imaging mode 1; TXI‐2; texture and color enhancement imaging mode 2.

< Evaluation of the total score of 5 trainees/experts >

→≧20: improved, 11–19: equivalent, ≦10: depressed.

< Evaluation of the total score of 10 endoscopists>

→ ≧40: improved, 21～39: equivalent, ≦20: depressed.

Statistical analysis; The differences between trainees and experts for visibility scores were examined using the Mann‐Whitney U test.

Table 4 presents visibility scores by histological type. Regarding OGA/GA‐FG, the total visibility score was 44.0 for TXI‐1, 40.0 for TXI‐2, and 31.0 for NBI. For experts, these scores were 21.0, 19.0, and 15.0, respectively, whereas those for trainees were 23.0, 21.0, and 14.0, respectively. Using TXI‐1 and TXI‐2, the visibility score was significantly higher for trainees than for experts (*p* < 0.05). No significant difference in the visibility score was observed between trainees and experts for NBI (*p* = 0.130). For all endoscopists, total visibility scores of GA‐FGM were 45.0, 39.0, and 31.0 for TXI‐1, TXI‐2, and NBI, respectively. These scores for the experts were 21.0, 18.0, and 16.0, respectively, whereas those for the trainees were 24.0, 21.0, and 15.0, respectively. Using TXI‐2, the visibility score was significantly higher for trainees than for experts (*p *< 0.05). No significant differences were observed between trainees and experts for TXI‐1 and NBI (*p* = 0.167 and *p* = 0.829, respectively).

**TABLE 4:** Visibility scores in histological types.

		Score			*p‐*value
Histological type	Modality	All endoscopists (*n* = 10)	Experts (*n* = 5)	Trainees (*n* = 5)	Experts vs. Trainees
OGA/GA‐FG	Mode 1	44.0 ± 3.9	21.0 ± 2.2	23.0 ± 2.0	<0.05
	Mode 2	40.0 ± 3.7	19.0 ± 2.1	21.0 ± 1.9	<0.01
	NBI	31.0 ± 6.0	15.0 ± 2.8	14.0 ± 3.6	0.13
GA‐FGM	Mode 1	45.0 ± 2.9	21.0 ± 1.8	24.0 ± 1.6	0.167
	Mode 2	39.0 ± 3.0	18.0 ± 1.8	21.0 ± 1.3	<0.05
	NBI	31.0 ± 4.0	16.0 ± 2.7	15.0 ± 1.4	0.829

Abbreviations: GA‐FG, gastric adenocarcinoma of fundic‐gland type; GA‐FGM, gastric adenocarcinoma of fundic‐gland mucosa type; OGA, oxyntic gland adenoma.

< Evaluation of the total score of 5 trainees/experts >

→≧20: improved, 11～19: equivalent, ≦10: depressed.

< Evaluation of the total score of 10 endoscopists>

→ ≧40: improved, 21～39: equivalent, ≦20: depressed.

Statistical analysis; The differences between trainees and experts for visibility scores were examined using the Mann‐Whitney U test.

### Interrater reliability

Table 5 presents the evaluation of interrater reliability among experts, trainees, and all endoscopists for TXI. The ICCs for TXI‐1 and TXI‐2 were 0.83 and 0.79 for all endoscopists, 0.65 and 0.56 for trainees, and 0.77 and 0.73 for experts, respectively. For NBI, the ICC was 0.86 for all endoscopists, 0.76 for trainees, and 0.76 for experts, respectively.

**TABLE 5:** Evaluation of texture and color enhancement imaging for interrater reliability of experts, trainees, and all endoscopists.

	Score		
GEN‐FGML	All endoscopists (*n* = 10)	Experts (*n* = 5)	Trainees (*n* = 5)
ICC (2.1)			
**Mode 1**	0.83	0.77	0.65
**Mode 2**	0.79	0.73	0.56
**NBI**	0.86	0.76	0.76

ICC: intraclass correlation coefficients	
ICC (2.1)	Criteria
0–0.2	Slight
0.21–0.4	Fair
0.41–0.6	Moderate
0.61–0.8	Substantial
0.81–	Almost perfect

Abbreviation: GEN‐FGML, gastric epithelial neoplasm of fundic‐gland mucosa lineage; ICC, intraclass correlation coefficients.

### Objective evaluations

**FIGURE 4:**
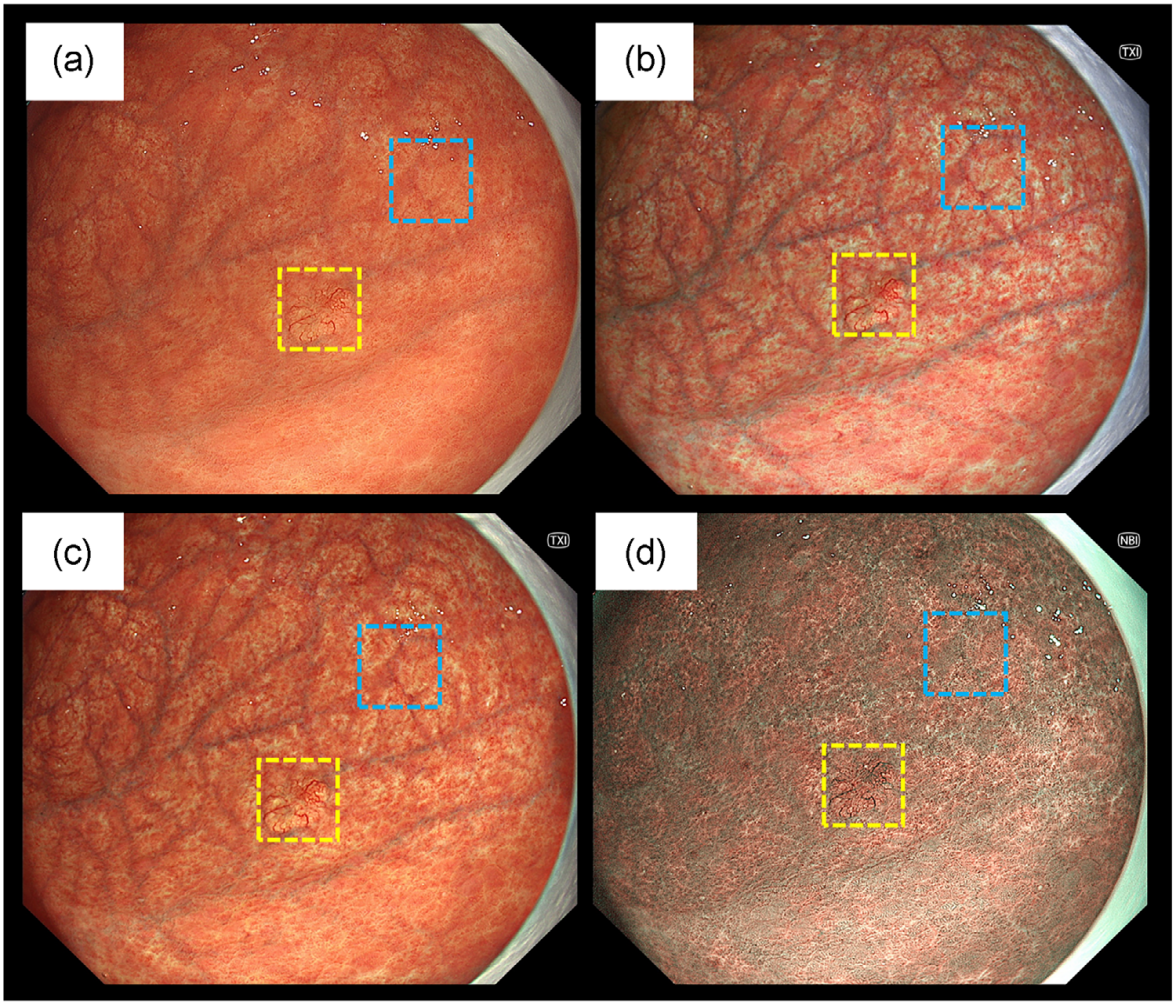
Representative endoscopic images using (a) white light imaging, (b) texture and color enhancement imaging mode 1, (c) texture and color enhancement imaging mode 2, and (d) narrow‐band imaging with the region of interest (40 × 40 pixels). Regions of interest were set in tumor (yellow box) and background gastric mucosa (blue box) and the same positions in all three images of a particular lesion were selected.

Figure 4 presents the representative endoscopic images and ROIs. The L* a* b* color values of the lesion (yellow box) and background mucosa (blue box) were calculated. Table 6 presents the results of the objective evaluations using the L*, a*, and b* color values. There were significant differences between TXI‐1 and WLI for the L*, a*, and b* values of the lesion and the a* and b* values of the background mucosa (*p* < 0.01). The L* value of the lesion significantly differed between TXI‐2 and WLI (*p* < 0.01).

**TABLE 6:** Objective evaluations using L*, a*, and b* color values.

				*p‐*Value		
L* a* b* values	WLI	TXI‐1	TXI‐2	WLI vs. TXI‐1	WLI vs. TXI‐2	TXI‐1 vs. TXI‐2
**Lesion**						
L*	62.3 ± 6.1	65.9 ± 6.4	65.5 ± 6.5	<0.01	<0.01	0.627
a*	39.5 ± 6.7	33.1 ± 11.3	38.3 ± 7.9	<0.01	0.72	0.054
b*	40.7 ± 2.5	30.7 ± 4.0	41.5 ± 2.8	<0.01	0.13	<0.01
**Background mucosa**						
L*	63.6 ± 6.2	63.7 ± 7.1	62.9 ± 6.6	0.97	0.59	0.647
a*	38.3 ± 5.1	33.4 ± 6.0	38.9 ± 4.2	<0.01	0.62	<0.01
b*	40.4 ± 3.0	30.1 ± 3.3	40.5 ± 2.9	<0.01	0.62	<0.01

Abbreviations: TXI‐1, texture and color enhancement imaging mode 1; TXI‐2, texture and color enhancement imaging mode 2; WLI, white light imaging.

Statistical analysis; The differences between each image for L*, a*, and b* color values were examined using the Mann‐Whitney U test.

Table 7 presents the results of the objective evaluation using the color difference (ΔE*) based on the L* a* b* color spaces. ΔE* between the lesion and background gastric mucosa significantly differed between WLI and TXI‐1 (*p* < 0.01) and between WLI and TXI‐2 (*p* < 0.01). No significant difference was observed between WLI and NBI (*p* = 0.60).

**TABLE 7:** Objective evaluations using the color difference.

L*a*b* values					*p*‐value			
	WLI	TXI‐1	TXI‐2	NBI	**WLI vs. TXI‐1**	**WLI vs. TXI‐2**	**WLI vs. NBI**	**TXI‐1 vs. TXI‐2**
ΔE*	7.3 ± 5.3	13.8 ± 8.0	10.4 ± 6.7	7.8 ± 5.3	<0.01	<0.01	0.60	0.03

Abbreviations: NBI, narrow‐band imaging; TXI‐1, texture and color enhancement imaging mode 1; TXI‐2, texture and color enhancement imaging mode 2; WLI: white light imaging.

Statistical analysis; The differences between each image for color difference (ΔE*) were examined using the Mann‐Whitney U test.

## DISCUSSION

This study is the first to examine the usefulness of TXI for the visibility of GEN‐FGML. The total visibility scores for all endoscopists were significantly higher for TXI than for WLI. The inter‐rater reliability for TXI‐1 was “almost perfect” for all endoscopists. We concluded that TXI, especially TXI‐1, improved the visibility of GEN‐FGML compared with WLI, as TXI‐1 displayed good separation of the color distribution, and significant differences between WLI and TXI‐1 were observed for color differences in the objective evaluation with the L* a* b* color values and ΔE*.

WLI is divided into a texture component and a base component with a brightness component. The images of each component are then properly processed and synthesized as TXI‐2. TXI‐1 is the image that proceeds color tone enhancement to TXI‐2. In principle, TXI‐2 enhances texture and brightness, and TXI‐1 further enhances color. Because the color contrast between red and white is greater under TXI‐1 than under TXI‐2, TXI‐1 might improve the visibility of GEN‐FGMLs regardless of the endoscopist's diagnostic skills. Therefore, TXI‐1 might be useful for detecting GEN‐FGMLs in screening EGD.

In the L* a* b* analysis in this study, TXI provided good separation of the color distribution compared with WLI. In addition, ΔE* significantly differed between WLI and TXI. Several studies conducted pigment analysis of EGC using TXI.^16^, ^17^, ^18^ Ishikawa et al. investigated the color difference between the non‐neoplastic and neoplastic areas of 12 gastric neoplasms (carcinoma and adenoma), finding that ΔE* was higher for TXI‐1 than for TXI‐2.^16^ Shijimaya et al. also assessed the visibility of EGC using subjective scores and ΔE*. They revealed that TXI‐1 enhanced EGC lesions and proved effective for EGC detection after *H. pylori* eradication, especially among trainee endoscopists.^18^ In summary, previous studies reported that color differences and visibility scores are highest for TXI‐1, suggesting that this technology might increase the detection of EGC in patients undergoing screening EGD. By contrast, NBI did not improve the visibility of GEN‐FGML in our study. NBI emphasizes microvessels and the microsurface architecture, making it useful for the endoscopic diagnosis of EGC. However, NBI did not enhance color contrast and texture because the surface layers of GEN‐FGML are often covered by non‐neoplastic epithelial mucosa.

Concerning histopathological types, TXI improved the visibility of OGA/GA‐FG and GA‐FGM. Regarding OGA/GA‐FG, three of four features on WLI; SMT shape, whitish color, and dilated vessels with branching architecture, have been attributed to the growth pattern of the tumor under the non‐neoplastic epithelium covering the surface layer.^11^ In this study, 80% of OGA/GAFG lesions were whitish in color. Therefore, TXI improved the visibility of GA‐FG because the contrast of coloration is more emphasized compared with WLI. Regarding GA‐FGM, neoplastic cells differentiating toward the foveolar epithelium were exposed on the superficial layer of the tumor, and neoplastic cells similar to fundic gland cells continuously invaded from the portion exposed on the surface to the deep layer of the tumor.^11^ Therefore, TXI improved the visibility of GA‐FGM as a typical EGC because the contrast of the surface structure is more emphasized compared with WLI. Additionally, TXI improved the visibility of Type 3 lesions because the contrast of coloration is more emphasized compared with WLI, similarly as observed for OGA/GA‐FG. In summary, TXI might be useful for the detection of GEN‐FGML because TXI emphasizes the whitish color of OGA/GA‐FG and GA‐FGM and the surface structure of GA‐FGM.

In the comparison of visibility for GEN‐FGML between experts and trainees, trainees had a significantly higher score than experts using TXI. In the analysis of histological type, trainees had a significantly higher score than experts for OGA/GA‐FG using TXI and a significantly higher score for GA‐FGM using TXI‐2. These results revealed that experts could capture the endoscopic features of GEN‐FGML using WLI alone because of their extensive experience in the endoscopic diagnosis of GEN‐FGML using WLI.

Ishibashi et al. found that HPUGCs such as GEN‐FGML with different endoscopic features than typical gastric cancer are difficult to diagnose without a thorough understanding of their endoscopic characteristics. Because HPUGC is often missed during endoscopic screening, it is important to educate endoscopists about the identifying features of HPUGC.^14^ Knowledge of endoscopic features and experience in diagnosis are necessary to detect rare cancers such as GEN‐FGML. Therefore, this study might help endoscopists identify the endoscopic features of GEN‐FGML, and TXI might be useful for GEN‐FGML detection compared with WLI regardless of the endoscopist's diagnostic skills.

Multiple limitations of this study must be addressed. First, a small number of patients were enrolled from a single center. Second, because one expert (Yoichi Akazawa) both performed the endoscopic examination and evaluated the endoscopic images of GEN‐FGML, the subjective assessment of visibility possibly might have resulted in observer bias. To address this issue, we conducted an objective quantitative analysis using ΔE* for each image. Finally, we examined visibility, but not detectability, in images obtained from daily practice. Therefore, well‐designed large‐scale clinical trials for the detection of GEN‐FGML are warranted to investigate the performance of TXI during real‐time EGC screening.

In conclusion, TXI, especially TXI‐1, improved the visibility of GEN‐FGML for all endoscopists compared with WLI in both subjective and objective evaluations.

## CONFLICT OF INTEREST STATEMENT

None.

## ETHICS STATEMENT

The present study was approved by the ethics committee of Juntendo University Hospital (No. 20–347) and performed according to the tenets of the Declaration of Helsinki.

## PATIENT CONSENT STATEMENT

All patients provided written informed consent to participate in this study.

## CLINICAL TRIAL REGISTRATION

The study protocol was registered in the University Hospital Medical Information Network Clinical Trials Registry (UMIN000045323).

## Supporting information




**Figure S1**: *H. pylori*‐uninfected status was defined as per the following four criteria. Patients who met all four criteria were defined as *H. pylori*‐uninfected.

**Figure S2**: Immunohistochemical classification of GEN‐FGML. According to the histopathological classification, GEN‐FGML was classified into OGA, GA‐FG and GA‐FGM.

**Figure S3**: Histopathological classification of GA‐FGM. According to the mucosal architecture of foveolar epithelium and the fundic gland, GA‐FGM can be further classified into 3 subtypes.
