# Supplementary figures and images for: Visibility evaluation of gastric epithelial neoplasm of fundic gland mucosa lineage using texture and color enhancement imaging
Source: DEN Open. 2025 Apr 8;5(1):e70110. doi: 10.1002/deo2.70110 (PMC11977659; doi:10.1002/deo2.70110)

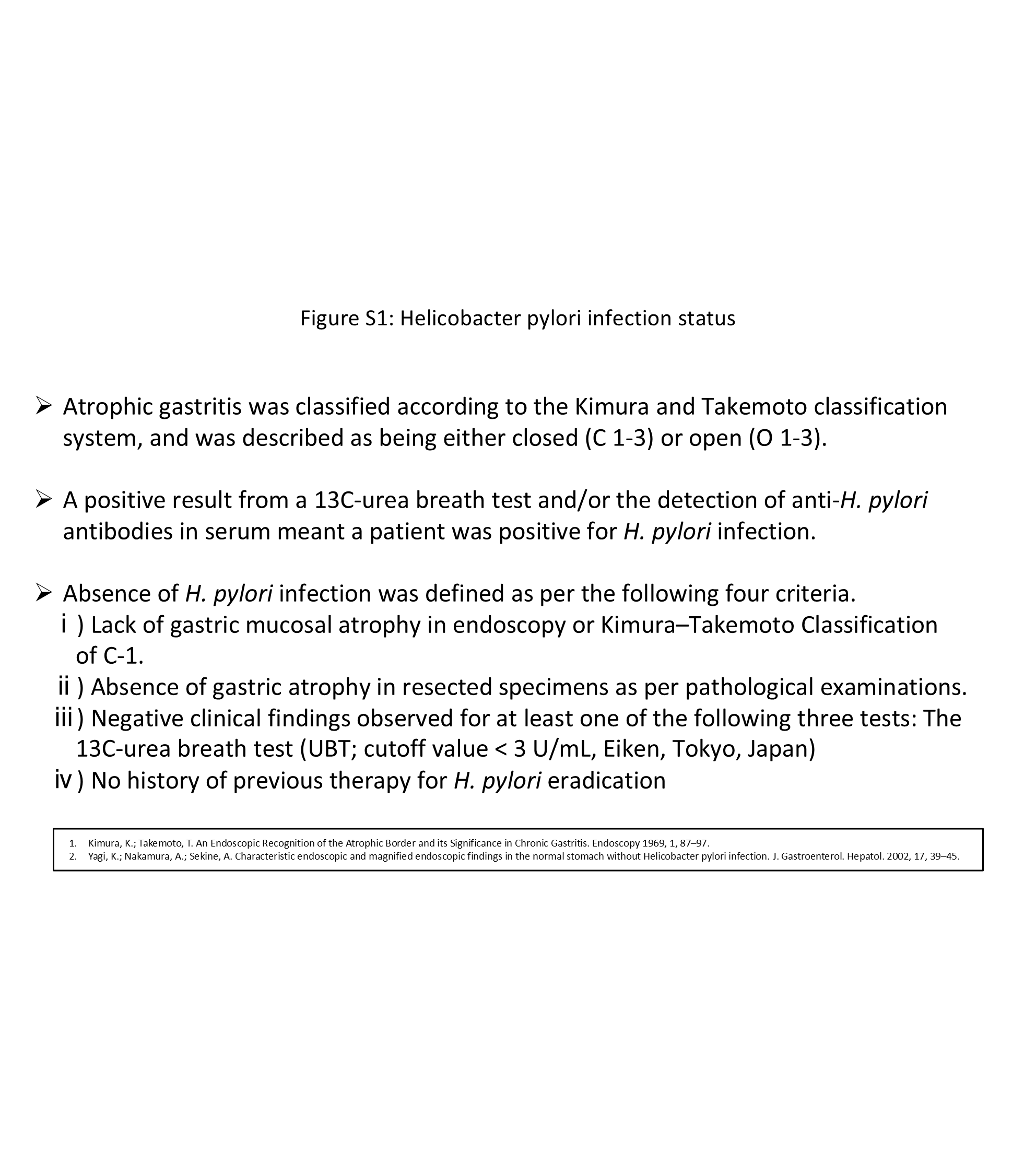

Supplement: Supplementary file 1 — Figure S1: H. pylori‐uninfected status was defined as per the following four criteria. Patients who met all four criteria were defined as H. pylori‐uninfected. [file DEO2-5-e70110-s002.tif]

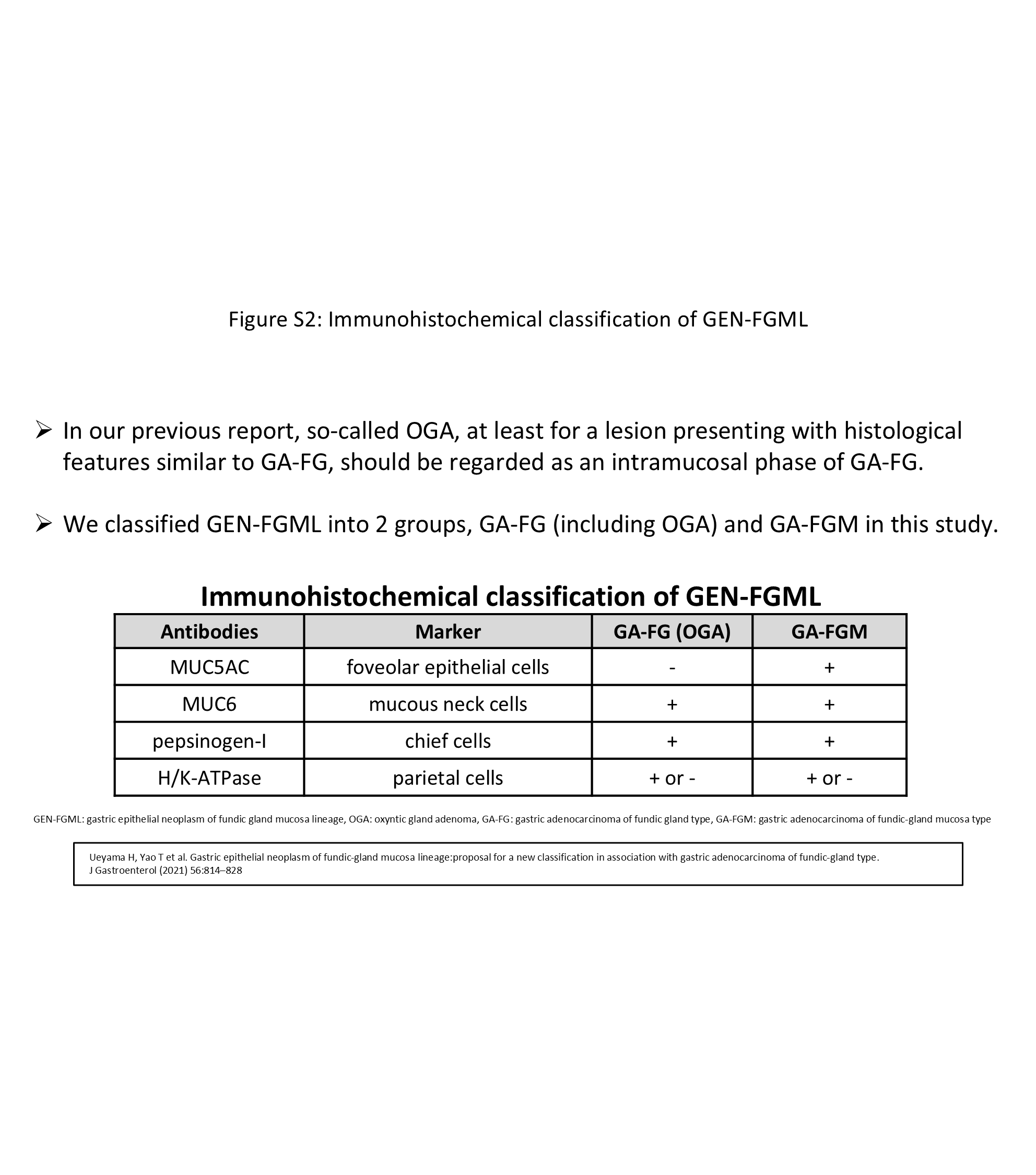

Supplement: Supplementary file 2 — Figure S2: Immunohistochemical classification of GEN‐FGML. According to the histopathological classification, GEN‐FGML was classified into OGA, GA‐FG and GA‐FGM. [file DEO2-5-e70110-s003.tif]

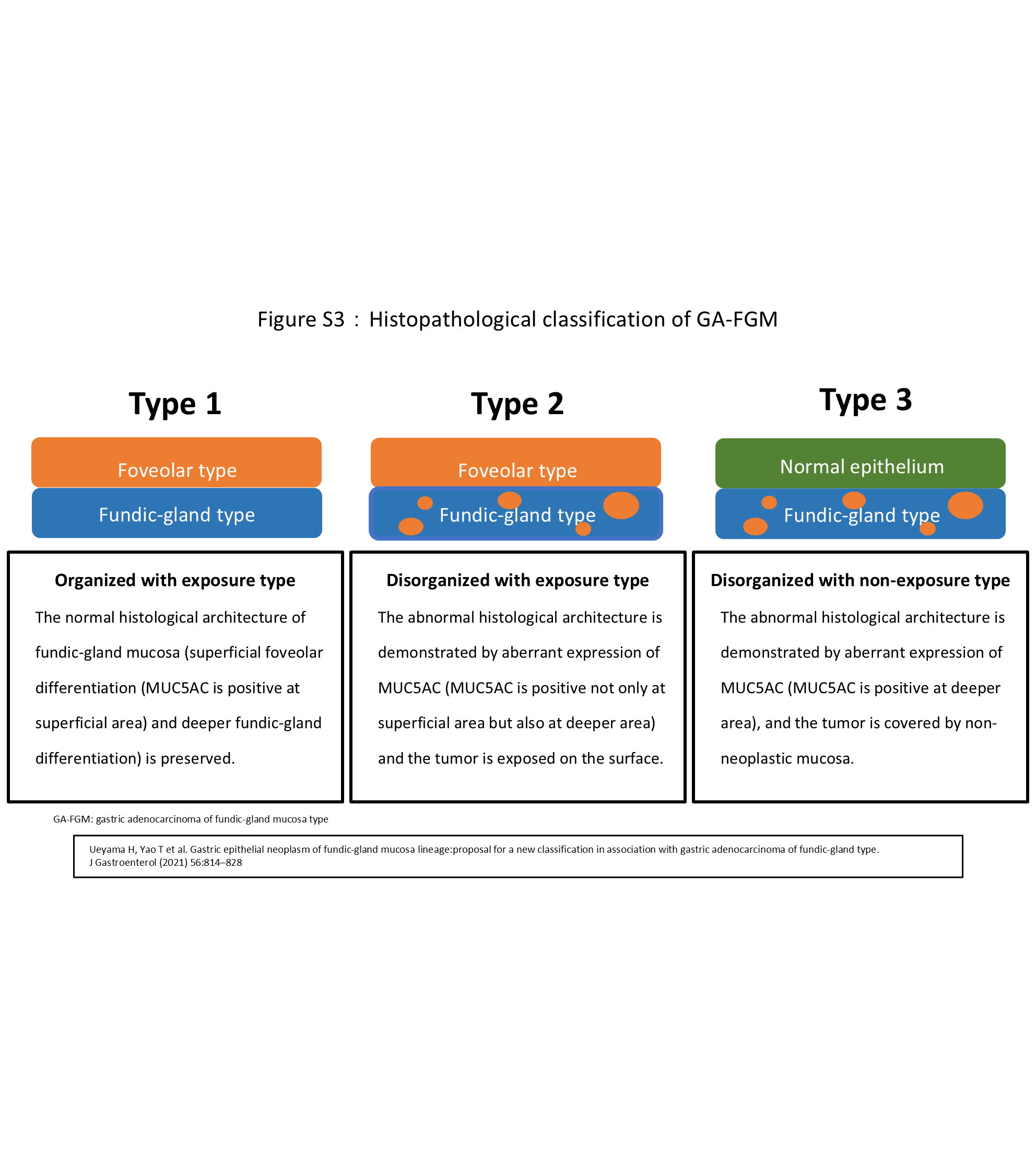

Supplement: Supplementary file 3 — Figure S3: Histopathological classification of GA‐FGM. According to the mucosal architecture of foveolar epithelium and the fundic gland, GA‐FGM can be further classified into 3 subtypes. [file DEO2-5-e70110-s001.tif]
